# Antiproliferative Activity of *N*-Acylhydrazone Derivative on Hepatocellular Carcinoma Cells Involves Transcriptional Regulation of Genes Required for G2/M Transition

**DOI:** 10.3390/biomedicines12040892

**Published:** 2024-04-18

**Authors:** Amanda Aparecida Ribeiro Andrade, Fernanda Pauli, Carolina Girotto Pressete, Bruno Zavan, João Adolfo Costa Hanemann, Marta Miyazawa, Rafael Fonseca, Ester Siqueira Caixeta, Julia Louise Moreira Nacif, Alexandre Ferro Aissa, Eliezer J. Barreiro, Marisa Ionta

**Affiliations:** 1 Institute of Biomedical Sciences, Federal University of Alfenas, Alfenas 37130-001, MG, Brazilaissa@unifesp.br (A.F.A.); 2 Institute of Chemistry, Fluminense Federal University, Niterói 24020-140, RJ, Brazil; 3 School of Dentistry, Federal University of Alfenas, Alfenas 37130-001, MG, Brazil; 4 Laboratory of Evaluation and Synthesis of Bioactive Substances (LASSBio), Institute of Biomedical Sciences, Federal University of Rio de Janeiro, Rio de Janeiro 21941-914, RJ, Brazil

**Keywords:** liver, anticancer, antiproliferative, cell cycle, apoptosis, gene expression

## Abstract

Liver cancer is the second leading cause of cancer-related death in males. It is estimated that approximately one million deaths will occur by 2030 due to hepatic cancer. Hepatocellular carcinoma (HCC) is the most prevalent primary liver cancer subtype and is commonly diagnosed at an advanced stage. The drug arsenal used in systemic therapy for HCC is very limited. Multikinase inhibitors sorafenib (Nexavar^®^) and lenvatinib (Lenvima^®^) have been used as first-line drugs with modest therapeutic effects. In this scenario, it is imperative to search for new therapeutic strategies for HCC. Herein, the antiproliferative activity of *N*-acylhydrazone derivatives was evaluated on HCC cells (HepG2 and Hep3B), which were chemically planned on the ALL-993 scaffold, a potent inhibitor of vascular endothelial growth factor 2 (VEGFR2). The substances efficiently reduced the viability of HCC cells, and the **LASSBio-2052** derivative was the most effective. Further, we demonstrated that **LASSBio-2052** treatment induced *FOXM1* downregulation, which compromises the transcriptional activation of genes required for G2/M transition, such as *AURKA* and *AURKB*, *PLK1*, and *CDK1*. In addition, **LASSBio-2052** significantly reduced *CCNB1* and *CCND1* expression in HCC cells. Our findings indicate that **LASSBio-2052** is a promising prototype for further in vivo studies.

## 1. Introduction

Liver cancer is the second and third leading cause of cancer-related deaths, respectively, in the male and female sexes [1]. It is estimated that approximately one million deaths will occur by 2030 due to this type of cancer. Hepatocellular carcinoma (HCC) is the most common subtype of primary liver cancer (around 75–85% of all diagnosed cases) [2].

Hepatocarcinogenesis is an intricate process characterized by multiple stages marked by genetic and epigenetic alterations. Key events in the development of HCC include chronic inflammation, heightened cell proliferation, resistance to apoptosis, and the emergence of tumor stem cells [2].

The risk factors for HCC are well-established and include chronic viral infection (Hepatitis B or Hepatitis C), excessive alcohol consumption, and aflatoxin B1 uptake. Importantly, non-alcoholic fatty liver disease (NAFLD) and non-alcoholic steatohepatitis (NASH) have become significant risk factors for HCC in recent years [3,4].

Commonly, HCC is diagnosed at an advanced stage, and the patients are treated with systemic therapy. The multikinase inhibitors sorafenib (Nexavar^®^) and lenvatinib (Lenvima^®^) have been used as first-line treatments for advanced HCC with modest therapeutic responses [2].

As of 2018, additional protein kinase inhibitors and monoclonal antibodies have received approval as second-line therapeutics for patients resistant to sorafenib, encompassing regorafenib (Stivarga^®^), cabozantinib (Cabometyx^®^), ramucirumab (Cyramza^®^), and nivolumab (Opdivo^®^). Regorafenib and cabozatinib function as protein kinase inhibitors, while ramucirumab and nivolumab act as monoclonal antibodies targeting VEGFR2 and PD-1, respectively [2,5]. In accordance with findings by Chagas et al. (2020) [6], the utilization of anti-VEGFR2 antibodies demonstrated efficacy in enhancing the survival rates of patients previously treated with sorafenib.

In addition to the gene pathways targeted by VEGFR2 inhibitors, advancements in cancer treatment could also involve pathways related to the cell cycle [7]. These genes play a pivotal role in regulating cell growth and division, making them a significant focal point. The inhibition of key cell cycle genes, such as aurora kinases and cyclins, holds the potential to halt the uncontrolled proliferation of cancer cells [8,9,10]. This approach is noteworthy for its potential to minimize the side effects commonly associated with traditional treatments that often impact healthy cells. By specifically targeting the highly active cell cycle genes prevalent in rapidly proliferating cancer cells, treatments can become more precise and effective [7,11]. This strategy represents a promising avenue for combating cancer in a personalized and accurate manner.

Despite the introduction of new drugs for the treatment of HCC, the 5-year survival rate is still very low, corresponding to 10% for local tumors and 3% for metastatic tumors [12,13], which motivates the constant search for new agents capable of improving therapeutic proposals for HCC.

Therefore, it is crucial to search for new drug candidates for HCC. The purpose of this study was to evaluate the effects of *N*-acylhydrazone derivatives (**LASSBio-2027**, **LASSBio-2029**, and **LASSBio-2052**) on the proliferative behavior of hepatocarcinoma cells. These derivatives were chemically planned on the ALL-993 scaffold, which is a potent inhibitor of vascular endothelial growth factor 2 (VEGFR2) [14]. For this purpose, we utilized HepG2 and Hep3B cells, which are widely employed in cancer research due to their distinct characteristics. HepG2 cells typically express *TP53*, whereas Hep3B cells lack both *TP53* alleles. Additionally, HepG2 cells do not harbor the integrated hepatitis B virus (HBV) genome, unlike Hep3B cells, which contain integrated HBV genome segments. These genetic differences between the cell lines can significantly influence their responses to various treatments, providing valuable insights for the development of more effective therapies against hepatocellular carcinoma [15].

The results indicate that all target derivatives, especially **LASSBio-2052**, effectively reduce HepG2 and Hep3B cell proliferation by modulating key regulators of the G2/M transition. This involves decreased mRNA levels of *AURKA*, *AURKB*, *PLK1*, *CDK1*, and *FOXM1*. 

## 2. Materials and Methods

### 2.1. Cell Lines and Culture Cell Conditions

Liver cancer cell lines (HepG2 and Hep3B) were used in the present study, which were purchased from the Rio de Janeiro Cell Bank. Primary fibroblasts (PFB), derived from human skin, were used as a non-transformed cell model. They were kindly provided by Prof. Dr. Angel Mauricio de Castro Gamero from the Institute of Nature Sciences at UNIFAL-MG (approved by the ethics committee—Process: 2.082. 524). Cell cultures were maintained in DMEM/F12 (Dulbecco’s Modified Eagle’s Medium plus F12, Sigma-Aldrich, St. Louis, MO, USA) supplemented with 10% fetal bovine serum (FBS, Cultilab, Campinas, SP, Brazil). Cells were grown in a humidified atmosphere of 95% air and 5% CO_2_ at 37 °C, and subcultures were performed regularly. The stocks were maintained in liquid nitrogen.

### 2.2. Synthesis of the N-Acylhydrazone Derivatives

**Figure 1:**
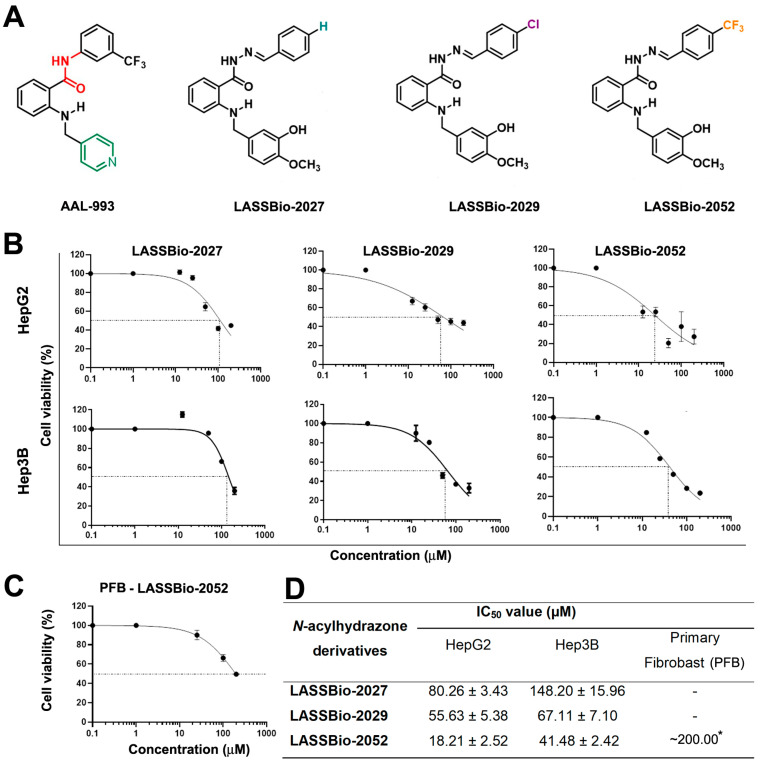
(**A**) Chemical structure of AAL-993 and *N*-acylhydrazone derivatives (NAH). (**B**,**C**) Dose–response curves. HepG2, Hep3B, and primary fibroblasts were treated for 48 h with NAH derivatives. (**D**) IC_50_ values were determined after 48 h treatment. * The IC_50_ for **LASSBio-2052** in human dermal primary fibroblasts (PFB) was estimated visually from the graph once its cytotoxic effect on normal cells was not sufficient to determine the exact IC_50_ value.

The substances **LASSBio-2027**, **LASSBio-2029**, and **LASSBio-2052** (Figure 1A) were synthesized and characterized as previously described by Pauli et al. (2020) [14].

For biological assays, the substances were solubilized in DMSO at 20 mM, and then diluted in culture medium immediately before treatment. The final concentration of DMSO in the culture medium was 0.1% (*v*/*v*) and did not affect cell viability under tested experimental conditions.

### 2.3. Cell Viability—Sulforhodamine B (SRB) Colorimetric Assay

Cell viability was determined by the SRB assay, which correlates cellular protein content with the viability rate [16]. HepG2, Hep3B, and PFB were seeded into 96-well plates (1 × 10^4^ cells/well). The cultures were treated for 48 h with the compounds **LASSBio-2027**, **LASSBio-2029**, and **LASSBio-2052** at different concentrations (0–200 μM) to determine the IC_50_ values. Cell monolayers were fixed with 10% (*w/v*) trichloroacetic acid at 4 °C for 1 h and stained with SRB (0.4% in 1% acetic acid) for 1 h. Next, the samples were washed repeatedly with 1% (*v/v*) acetic acid to remove unbound SRB. The protein-bound dye was dissolved in a 10 mM Tris-base solution (30 min) for optical density (OD) determination at 540 nm, using 690 nm as a reference value in a microplate reader. IC_50_ values were calculated using GraphPad Prism^®^ 8.0 software (GraphPad Software, Inc., San Diego, CA, USA).

In the next step, we evaluated the proliferation kinetics of HCC cells. For this, the cells were treated with **LASSBio-2052** at 10 or 20 μM (HepG2) and 20 or 40 μM (Hep3B), and viability was determined at 0, 24, and 48 h. The data are presented as mean ± standard deviation (SD) from at least three independent experiments.

### 2.4. Clonogenic Assay

To evaluate the long-term proliferation ability of HCC cells, a clonogenic assay was performed [17]. The cells were seeded at low density into 35-mm plates (1000 cells/plate). HepG2 cells were treated with **LASSBio-2052** at 10 or 20 μM for 48 h and recovered in a drug-free medium for 12 days. Hep3B cells were treated with **LASSBio-2052** at 20 or 40 μM for 48 h and recovered in a drug-free medium for 12 days. After the recovery time, the colonies were fixed with methanol for 30 min and stained with crystal violet for 5 min. The quantification of colonies was performed using a stereomicroscope (20× magnification). Only colonies with >50 cells were considered for analysis. The data are presented as the mean ± SD of three independent experiments.

### 2.5. Cell Cycle Analysis

The cell cycle distribution pattern was determined by flow cytometry. Cells were seeded into 35-mm Petri plates at a density of 1 × 10^5^ cells/plate. Cells were treated with **LASSBio-2052** at 10 or 20 μM (HepG2) and 20 or 40 μM (Hep3B) for 48 h. Afterward, the cells were collected by enzymatic digestion (Trypsin-EDTA solution, Sigma-Aldrich, St. Louis, MO, USA) and fixed with 75% ethanol at 4 °C overnight. Subsequently, the DNA was stained with a propidium iodide (PI) solution (90 μg/mL) containing RNase (2.5 mg/mL) for 1 h at 4 °C. The analysis was performed using a flow cytometer (Guava easyCyte 8HT, Hayward, CA, USA). The data are presented as the mean ± SD of three independent experiments.

### 2.6. Gene Expression Profile Determined by qPCR

Expression of the target genes (*CCNB1*, *CCND1*, *CDKN1A*, *CDK1*, *PLK1*, *FOXM1*, *AURKA*, and *AURKB*) was evaluated by real-time PCR (RT-qPCR). The sequences of primers are shown in Table S1. Briefly, cells were seeded into 35-mm Petri plates at a density of 2 × 10^5^ cells/plate. Cells were treated with **LASSBio-2052** at 10 or 20 μM (HepG2) and 20 or 40 μM (Hep3B). After 24 h of treatment, the cells were collected by enzymatic digestion, and total RNA was extracted using the RNeasy^®^ Mini kit (Qiagen, Mississauga, ON, Canada). Total RNA concentrations were measured by a spectrophotometer using a NanoDrop^®^ ND 1000 (NanoDrop Technologies, Wilmington, DE, USA). Then, 1 µg of total RNA was incubated with DNase (1 U; ThermoFisher, Waltham, MA, USA) and subjected to reverse transcription (RT) using the High-Capacity cDNA Reverse Transcription Kit^®^ (ThermoFisher). Relative quantification of mRNA was performed as previously described [18,19]. The data are presented as the mean ± SEM of four independent experiments. The raw cycle threshold values of the samples are in Table S2.

### 2.7. Apoptosis Evaluation

Apoptosis was evaluated by phosphatidylserine externalization detection using the Annexin V-FITC/PI Kit (#V13242, Invitrogen, Waltham, MA, USA). Cells were seeded into 35-mm Petri plates at a density of 1 × 10^5^ cells/plate. Cells were treated with **LASSBio-2052** at 10 or 20 μM (HepG2) and 20 or 40 μM (Hep3B) for 48 h. The cells were collected by enzymatic digestion (Trypsin/EDTA, Sigma-Aldrich) and centrifuged at 200× *g* for 5 min at 4 °C. The samples were washed with ice-cold PBS, and 100 μL of a mixture solution containing buffered Annexin V- FITC and PI was added. After 20 min of incubation at room temperature in a dark chamber, the samples were analyzed by flow cytometry using GuavaSoft 2.7 software. The data are presented as the mean ± SD of three independent experiments.

### 2.8. Survival Analysis of HCC Patients

We plotted the overall survival probability of HCC patients using Kaplan–Meier plots by separating the samples into two groups (patients with high and low expression of each gene altered by **LASSBio-2052**). We used gene expression and survival data from The Cancer Genome Atlas (TCGA). Specifically, we generated plots for *AURKA*, *AURKB*, *PLK1*, *CDK1*, *CCNB1*, *CCND1*, *FOXM1*, and *CDKN1A*. We also generate Kaplan–Meier plots composed of the average expression of all the combined genes downregulated by **LASSBio-2052** (*AURKA*, *AURKB*, *PLK1*, *CDK1*, *CCNB1*, *CCND1*, and *FOXM1)* which we named Signature. The mean expression of the Signature was calculated per TCGA sample. For the *CDKN1A* gene, in addition to evaluating it in the same way as the other genes in all patients, we also performed an analysis separating patients with wild-type *TP53* and patients with mutated *TP53*, resulting in three analyses for this gene. The samples were split by median groups representing low and high expression, and log-rank *p*-values were displayed. The plots were generated with the ‘survminer’ version 0.4.9 [20] package in R Statistical Software v4.1.0 [21]. Molecular profiles and patient metadata were obtained using the package “cBioPortalData” v2.6.1 [22], using studyId = “lihc_tcga” and molecularProfileId = “lihc_tcga_rna_seq_v2_mrna_median_all_sample_Zscores”.

### 2.9. Statistical Analysis

The results were tested for significance using one-way analysis of variance (ANOVA), followed by a Dunnett post-test using GraphPad Prism^®^ 8.0 software. *p*-values < 0.05 were considered statistically significant.

## 3. Results

### 3.1. **LASSBio-2052** Has Antiproliferative Activity on Hepatocarcinoma Cells

The chemical structures of the three *N*-acylhydrazone derivatives developed from the original ALL-993 molecule differ only concerning the substituents on the aryl group (Figure 1A). To test the effect of these different substituents on HCC cells, we evaluated the viability of HepG2 and Hep3B cells treated with different concentrations of the molecules. Following a 48 h treatment, there was a reduction in cell viability for all compounds tested; notably, **LASSBio-2052** exhibited the highest efficacy (Figure 1B,D). The determined IC_50_ values for **LASSBio-2052** were approximately 20 and 40 µM on HepG2 and Hep3B cells, respectively (Figure 1D). Additionally, **LASSBio-2052** underwent evaluation in human dermal primary fibroblasts (PFB) (Figure 1C). The IC_50_ for **LASSBio-2052** in PFB could not be ascertained with precision since the molecule was not cytotoxic enough to PFB (Figure 1D). Nonetheless, an estimation can be made from the graph, suggesting that the IC_50_ of **LASSBio-2052** in PFB was approximately 200 µM, greater than that observed in cancer cells.

**Figure 2:**
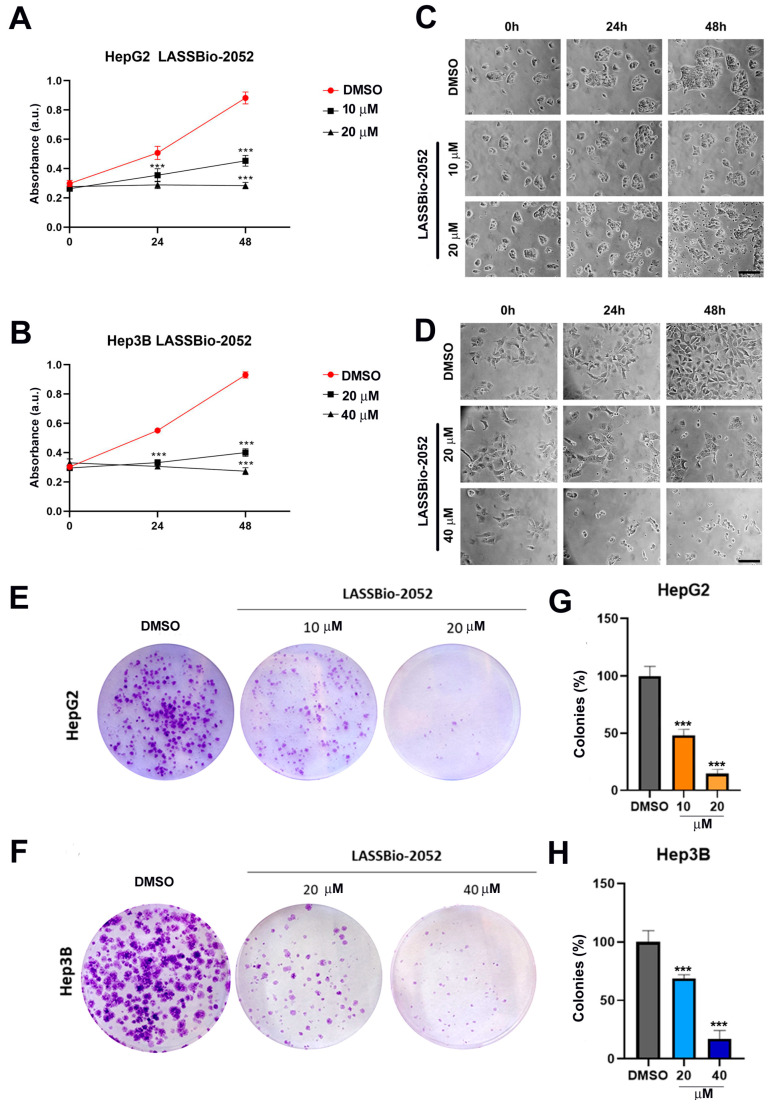
(**A**,**B**) Cell viability rate was determined in HepG2 (**C**) and Hep3B (**D**) cultures at 0, 24, and 48 h after treatment. (**C**,**D**) Representative images obtained by phase microscopy showing morphological aspects of cells. (**E**,**F**) Representative images from the clonogenic assay. The cells were treated for 48 h and recovered in fresh medium for 12 days. (**G**,**H**) Quantification of the number of colonies relative to control DMSO. *** *p <* 0.001 according to ANOVA followed by a Dunnett post-test.

We chose concentrations relative to the IC_50_ and IC_50_/2 of each cell type to evaluate the growth kinetics of cells at 0, 24, and 48 h after treatment (Figure 2A–D). IC_50_ concentrations (20 µM for HepG2; 40 µM for Hep3B) prevented growth and even reduced the number of cells in both cell types after 48 h (Figure 2A,B). Concentrations of IC_50_/2 (10 µM for HepG2; 20 µM for Hep3B) also prevented cell growth, but to a lesser extent (Figure 2A,B).

Photomicrographs of the cells at the same treatment time confirmed these results, showing that there was a lower confluence of cells after 48 h (Figure 2C,D). Furthermore, signs of cytotoxicity were evident in cultures treated with IC_50_ concentrations (20 µM for HepG2; 40 µM for Hep3B) (Figure 2C,D). Specifically, after 48 h of treatment, HepG2 cultures displayed cellular debris (Figure 2C), while Hep3B cultures exhibited rounded cells partially adhered to the substrate (Figure 2D).

The clonogenic assay confirmed these results, showing that **LASSBio-2052** was able to inhibit cell growth in the long term, evaluated after 12 days in cells previously treated for 48 h (Figure 2E–H).

### 3.2. **LASSBio-2052** Inhibits Cell Cycle Progression in Hepatocarcinoma Cells

**Figure 3:**
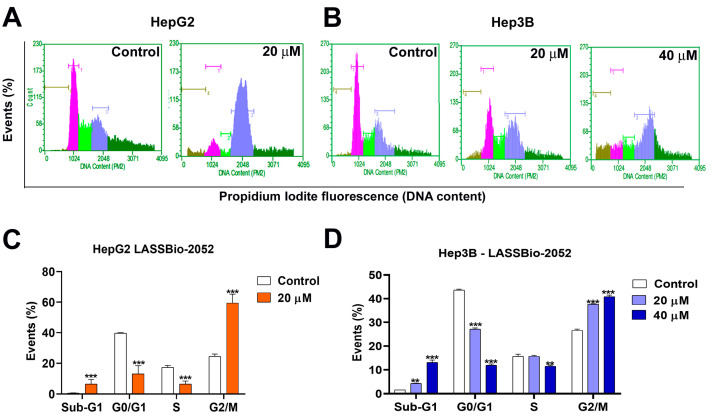
(**A**,**B**) Representative histograms obtained by flow cytometry after 48 h treatment with **LASSBio-2052**. (**C**,**D**) Cell cycle analysis. *** *p <* 0.001, ** *p <* 0.01 according to ANOVA followed by a Dunnett post-test.

We evaluated cell cycle progression to better understand the mechanism associated with the antiproliferative activity of **LASSBio-2052** on HepG2 and Hep3B cells (Figure 3A–D). The molecule inhibited cell cycle progression once there was an increase in G2/M population in both HepG2 (≈60%) (Figure 3A,C) and Hep3B (≈40%) cells (Figure 3B,D), treated with **LASSbio-2052** for 48 h compared with controls (≈25% for both HepG2 and Hep3B). Additionally, we observed an increased sub-G1 population in response to treatment in both cells, which is indicative of cell death.

### 3.3. **LASSBio-2052** Modulates Expression Profiles of Regulators of Cell Cycle 

**Figure 4:**
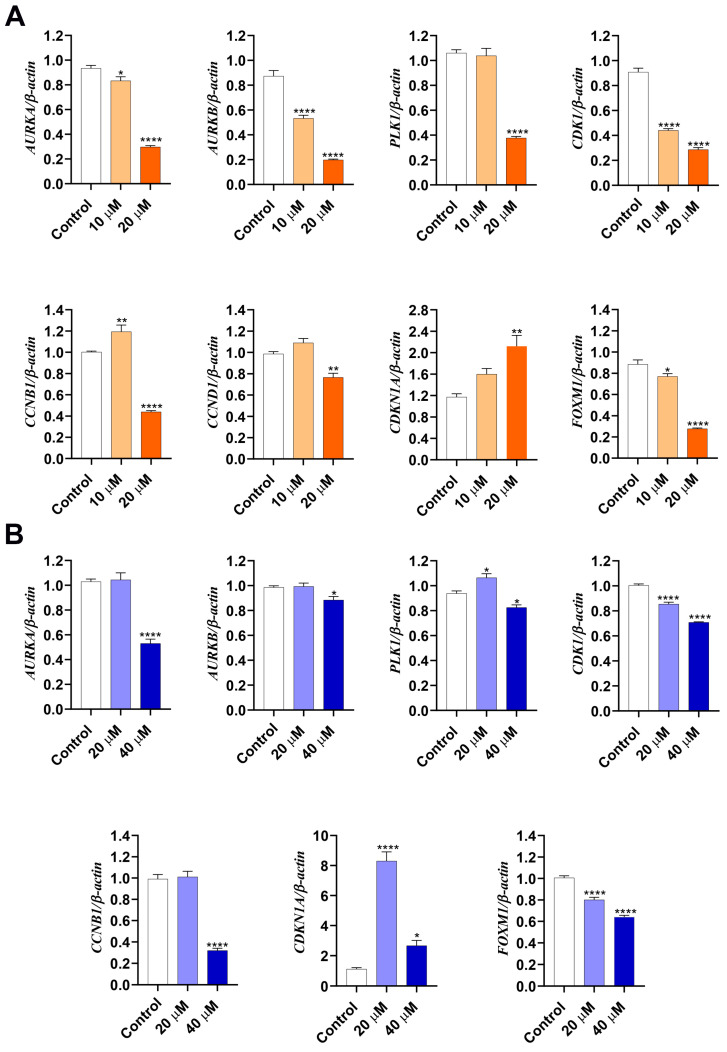
Gene expression profiles determined by RT-qPCR after 24 h treatment. (**A**) HepG2. (**B**) Hep3B. **** *p* < 0.0001, ** *p* < 0.01, * *p* < 0.05 according to ANOVA followed by a Dunnett post-test.

Based on cell cycle analysis, we evaluated the expression of genes associated with the regulation of the G2/M transition. Thus, we determined the expression of genes encoding mitotic kinases *CDK1*, *AURKA* (Aurora A), *AURKB* (Aurora B), and *PLK1*, cyclin-dependent kinase inhibitor *CDKN1A* (p21), and *CCNB1* (cyclin B). In both HepG2 and Hep3B, **LASSBio-2052** at 20 µM was able to reduce the mRNA abundance of all positive regulators of the cell cycle, such as *AURKA*, *AURKB*, *PLK1*, *CDK1*, and *CCNB1* (Figure 4A,B). On the contrary, the expression level of *CDKN1A* was significantly increased in both treated cell types (Figure 4A,B). For HepG2, we also evaluated the expression profile of *CCND1* (cyclin D1), which was also reduced in cells treated with 20 µM. Intriguingly, the lower concentration increased the expression of the *CCNB1* gene in HepG2 cells and the *PLK1* gene in Hep3B cells, while the higher concentration decreased the expression of these genes in the same cell type.

We also investigated the expression of *FOXM1*, a transcriptional activator involved in the M phase that regulates the expression of several cell cycle genes, such as *CCNB1* and *CCND1* [23]. We found that **LASSBio-2052** reduced the expression of *FOXM1* in a dose-dependent manner in both HepG2 (Figure 4A) and Hep3B cells (Figure 4B).

### 3.4. Cytotoxic Activity of **LASSBio-2052** on HCC Cells Involves Apoptosis Induction

**Figure 5:**
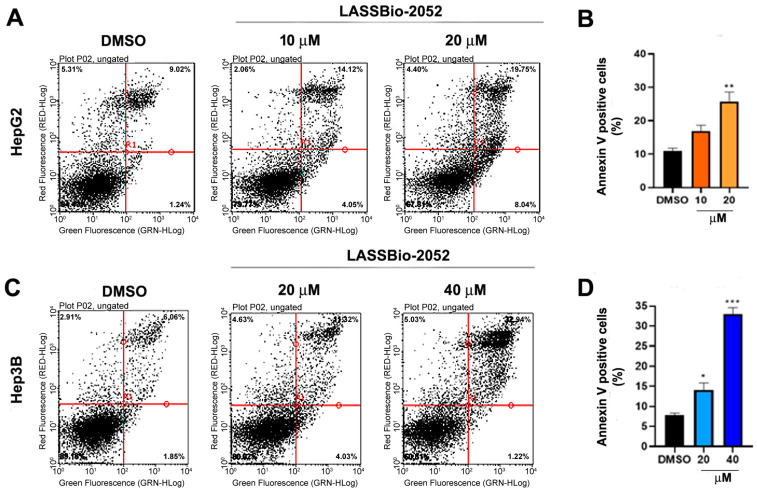
(**A**,**C**) Representative dot plots from the annexin assay. Cells were treated for 48 h with **LASSBio-2052**. (**B**,**D**) Determination of apoptotic cells considering the cell population positive for the Annexin V assay. * *p* < 0.05, ** *p* < 0.01, *** *p* < 0.001 according to ANOVA followed by a Dunnett post-test.

Since cell cycle analysis indicated an increase in sub-G1 population, an indication of cell death in response to treatment (Figure 3), we investigated the pro-apoptotic potential of **LASSBio-2052** on HepG2 and Hep3B cells using an annexin V assay (Figure 5A–D). Treating both cell types with the higher concentrations (IC_50_) increased the annexin-positive cells (Figure 5A–D), suggesting that **LASSBio-2052** can induce apoptotic cell death in HCC, especially in Hep3B cells. (Figure 5C,D).

### 3.5. Gene Expression Changes Induced by **LASSBio-2052** Are Associated with the Better Overall Survival of HCC Patients

**Figure 6:**
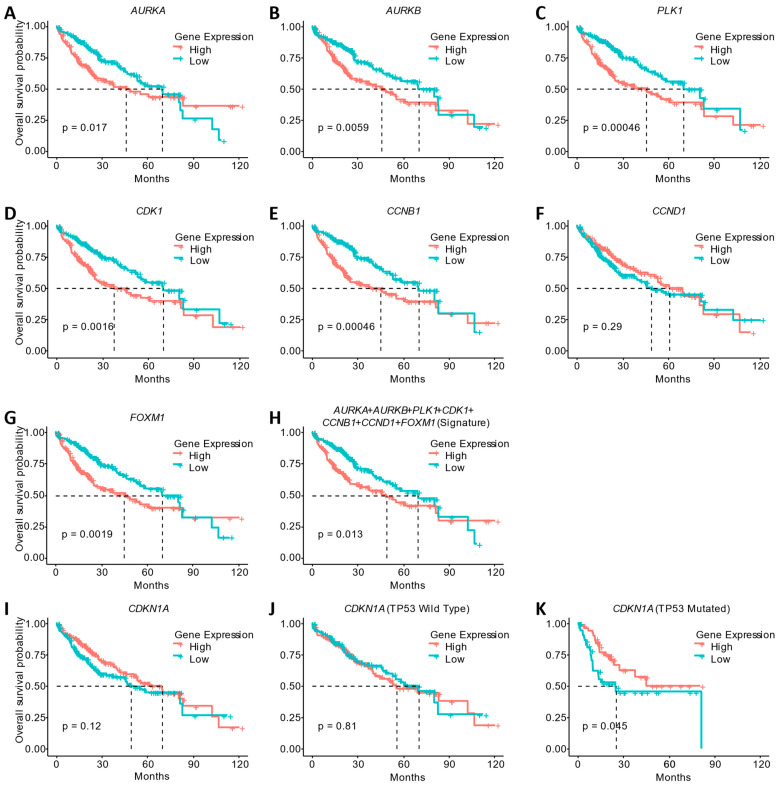
Reduced expression of genes downregulated by **LASSBio-2052** is associated with improved overall survival in patients with hepatocellular carcinoma. Overall survival probability analysis using samples from Liver Hepatocellular Carcinoma (TCGA, Firehose Legacy, study ID: “lihc_tcga”). (**A**–**G**) Single gene analysis. (**H**) Gene signature made with the average of all genes downregulated by **LASSBio-2052**. (**I**–**K**) *CDKN1A* analysis using the whole dataset (**I**), patients with *TP53* not mutated (**J**), and patients with *TP53* mutation (**K**). Log-rank *p*-values are presented.

**LASSBio-2052** was able to reduce the expression of genes related to the cell cycle. Therefore, we investigated whether the reduction in the expression of these genes has clinical relevance. For this, we utilized gene expression data from TCGA from patients with HCC (Figure 6). The reduction in the expression of the *AURKA* (Figure 6A), *AURKB* (Figure 6B), *PLK1* (Figure 6C), *CDK1* (Figure 6D), *CCNB1* (Figure 6E), and *FOXM1* (Figure 6G) genes, all downregulated by **LASSBio-2052**, is associated with better overall patient survival. *CCND1* expression was not associated with overall survival (Figure 6F, *p* > 0.05). When we combined all the genes, creating an average of the expression of all of them (Signature), the association was also significant. This suggests that, in addition to the expression of individual genes, the set of genes that was downregulated by **LASSBio-2052** is associated with better overall patient survival.

We also tested the expression of the *CDKN1A* tumor suppressor, the only one that was upregulated by **LASSBio-2052**. In this case, there was no statistically significant difference between gene expression and patient overall survival (Figure 6I). We then separated the patients according to mutations in the *TP53* gene. Patients with no mutation in *TP53* also did not show changes in overall survival according to *CDKN1A* expression (Figure 6J). However, patients with *TP53* mutations showed better overall survival when *CDKN1A* was upregulated (Figure 6K). This suggests that treatment with **LASSBio-2052** would be an interesting therapeutic approach, particularly for patients with *TP53* mutations, present in 30% of the patients analyzed.

## 4. Discussion

In the present study, we evaluated the antiproliferative activity of *N*-acylhydrazone derivatives, namely **LASSBio-2027**, **LASSBio-2029**, and **LASSBio-2052**. These compounds differ due to the presence of distinct groups at the aryl group. **LASSBio-2027** has a phenyl group, **LASSBio-2029** has a 4-chlorophenyl group, and the **LASSBio-2052** has the strongly electron withdrawing group trifluoromethyl (CF3) in the para-position of the phenyl group. Based on IC_50_ values, **LASSBio-2052** emerged as the most potent prototype when tested against hepatocellular carcinoma cells (HepG2 and Hep3B), in contrast to its previously reported effects on estrogen-positive breast cancer cells, where **LASSBio-2052** was not so cytotoxic to MCF-7 cells [18]. This discovery suggests that the presence of CF3 in **LASSBio-2052** holds significance for its cytotoxic activity and selectivity, specifically towards hepatocellular carcinoma cells. Interestingly, fluorine-containing compounds represent more than 50 percent of the best-selling drug molecules approved by the US Food and Drug Administration (FDA) [24].

**LASSBio-2052** effectively inhibited the proliferation of HepG2 and Hep3B cells in both short- and long-term scenarios, even when concentrations as low as the IC_50_ were employed. These findings are promising, given that both cell lines exhibit genetic alterations commonly observed in HCC, such as mutations in the *CTNNB1* gene and *TERT* promoter [2,25]. Additionally, the Hep3B cell line harbors parts of the hepatitis B virus (HBV) genome integrated [26] and does not express *TP53* due to deletion in both alleles [15]. Thus, the Hep3B cell line serves as a representative model for aggressive tumors, which are often resistant to treatment and associated with a poor prognosis [26,27,28]. Importantly, HBV integration is estimated to occur in 85–90% of HCCs associated with HBV, contributing to increased genetic damage and chromosomal instability in infected neoplastic cells [29].

Cell cycle analysis revealed an increase in G2/M and sub-G1 populations in both HCC cell lines following treatment with **LASSBio-2052**. These findings suggest that this compound is able to modulate cell cycle regulators and induce cell death. Moreover, we demonstrated that the elevated sub-G1 population was a result of apoptosis induction. Additionally, we assessed the gene expression profiles of positive and negative regulators of the cell cycle [11], considering the observed increase in the G2/M population induced by **LASSBio-2052**. The activation of mitotic kinases AURKA/B, CDK1, and PLK1 is crucial for G2/M transition and mitosis [30,31]. Consistent with previous data, RT-qPCR analysis revealed that **LASSBio-2052** led to the downregulation of genes encoding mitotic kinases (*AURKA*, *AURKB*, *PLK1*, and *CDK1*) in HepG2 and Hep3B cells. Furthermore, the relative expression of mRNA for *CCNB1* (cyclin B1) in both HepG2 and Hep3B and *CCND1* (cyclin D1) in HepG2 was significantly lower in **LASSBio-2052**-treated samples compared to control groups. 

Overexpression of AURKA/B, CDK1, and PLK1 has been reported in several malignant tumors, including HCC [30,32,33]. A recent study has demonstrated that the overexpression of AURKA, CDK1, and PLK1 is positively correlated with tumor grades and stage. Thereby, these genes may be used as prognostic biomarkers for HBV-induced HCC. Furthermore, there is a strong correlation between high expression of AURKA, CDK1, and PLK1 and the infiltration of immune cells [33]. The coordinated overexpression of FOXM1 and AURKA has been linked to the worst overall survival in sorafenib-treated patients with HCC [34]. 

While positive regulators of the cell cycle were downregulated by **LASSBio-2052** treatment, the negative regulator of the cell cycle, *CDKN1A* (encoding p21), was significantly upregulated. In Hep3B, the relative expression of this gene was approximately 8-fold higher in samples treated with **LASSBio-2052** at 20 µM, while in HepG2, the relative expression of *CDKN1A* was around 2-fold. These results are interesting because the transcriptional activation of *CDKN1A* typically occurs preferentially by TP53, and as mentioned before, Hep3B cells are TP53 deficient. Thus, the pronounced upregulation of *CDKN1A* in response to **LASSBio-2052** treatment in Hep3B cells likely occurred through TP53-independent pathways. It has been reported that the viral oncogenic protein HBx (expressed by Hep3B) inhibits the expression of *CDKN1A* at the transcriptional level by negatively regulating the SP1 transcriptional factor [35]. Hence, it is plausible that **LASSBio-2052** might have modulated the HBx/SP1 pathway. Alternatively, the elevated expression of *CDKN1A* in Hep3B might be a consequence of epigenetic regulation. It has been reported that *CDKN1A* gene transcription can be repressed by epigenetic mechanisms. Lou et al. (2019) [36] demonstrated that antisense lncRNAs, such as GATA3-AS1, promote cell proliferation and metastasis in HCC by suppressing proto-oncogenes, including *CDKN1A*. In gastric cancer, it was shown that *CDKN1A* was epigenetically silenced by the HOXA-AS2-EZH2 complex [37].

We also assessed the expression profile of *FOXM1*, a proliferation-associated transcription factor belonging to the forkhead box superfamily of proteins [38]. It has been reported that FOXM1 is overexpressed in HCC and sorafenib-resistant HCC samples. The overexpression of FOXM1 has been positively associated with a poor prognosis in HCC patients [39,40]. The ectopic expression of FOXM1 in HepG2 cells induced proliferation, while the opposite was observed when FOXM1 was silenced in Hep3B [39]. *FOXM1* knockdown also suppressed cell proliferation and induced G2/M cell cycle arrest in Huh7 cells [40]. The molecular mechanism by which FOXM1 is required for G2/M transition and mitosis onset involves the transcription activation of several genes, including mitotic kinases and cyclin B [41,42]. In the present study, we demonstrated that *FOXM1* and its downstream genes were downregulated by **LASSBio-2052** in both cell lines, reinforcing its antitumor potential in HCC. Our findings suggest that **LASSBio-2052** might be useful in treating tumors refractory to sorafenib.

## 5. Conclusions

In conclusion, our study focused on three *N*-acylhydrazone derivatives, with **LASSBio-2052** demonstrating the highest efficacy, reducing cell viability in both HepG2 and Hep3B HCC cells. Importantly, **LASSBio-2052** exhibited minimal cytotoxicity in human dermal primary fibroblasts. **LASSBio-2052** inhibited the growth kinetics of both cell types, as confirmed by short- and long-term assays demonstrating its inhibitory effect on HCC cell growth. Cell cycle analysis indicated G2/M arrest and an increased sub-G1 population, indicative of cell death, which was confirmed by increased apoptosis. Molecular analysis showed that **LASSBio-2052** downregulated key cell cycle regulators such as *AURKA*, *AURKB*, *PLK1*, *CDK1*, *CCNB1*, and *FOXM1* and upregulated *CDKN1A*. These changes in gene expression correlated with improved overall survival in patients with HCC. Additionally, upregulation of *CDKN1A* was positively associated with overall survival in patients with *TP53* mutations. In summary, our findings demonstrate that **LASSBio-2052** has promising antiproliferative and pro-apoptotic effects in HCC cells. Further in vivo investigations are warranted to validate these findings and explore **LASSBio-2052**’s translational implications in the treatment of HCC.

## Data Availability

Data are contained within the article and Supplementary Materials.

## Supplementary Materials

The following supporting information can be downloaded at: https://www.mdpi.com/article/10.3390/biomedicines12040892/s1, Table S1. Sequences of the primers used for amplification in real-time PCR; Table S2. Raw cycle threshold values obtained from real-time PCR.

## References

[1] Sung H., Ferlay J., Siegel R.L., Laversanne M., Soerjomataram I., Jemal A., Bray F. (2021). Global Cancer Statistics 2020: GLOBOCAN Estimates of Incidence and Mortality Worldwide for 36 Cancers in 185 Countries. CA A Cancer J. Clin..

[2] Villanueva A. (2019). Hepatocellular Carcinoma. N. Engl. J. Med..

[3] McGlynn K.A., Petrick J.L., El-Serag H.B. (2021). Epidemiology of Hepatocellular Carcinoma. Hepatology.

[4] Tümen D., Heumann P., Gülow K., Demirci C.-N., Cosma L.-S., Müller M., Kandulski A. (2022). Pathogenesis and Current Treatment Strategies of Hepatocellular Carcinoma. Biomedicines.

[5] Montironi C., Montal R., Llovet J.M. (2019). New Drugs Effective in the Systemic Treatment of Hepatocellular Carcinoma. Clin. Liver Dis..

[6] Chagas A.L., Mattos A.A.D., Carrilho F.J., Bittencourt P.L., Vezozzo D.C.P., Horvat N., Rocha M.D.S., Alves V.A.F., Coral G.P., Alvares-Da-Silva M.R. (2020). Brazilian society of hepatology updated recommendations for diagnosis and treatment of hepatocellular carcinoma. Arq. Gastroenterol..

[7] Lapenna S., Giordano A. (2009). Cell Cycle Kinases as Therapeutic Targets for Cancer. Nat. Rev. Drug Discov..

[8] Bolanos-Garcia V.M. (2005). Aurora Kinases. Int. J. Biochem. Cell Biol..

[9] Borah N.A., Reddy M.M. (2021). Aurora Kinase B Inhibition: A Potential Therapeutic Strategy for Cancer. Molecules.

[10] Hole S., Pedersen A.M., Lykkesfeldt A.E., Yde C.W. (2015). Aurora Kinase A and B as New Treatment Targets in Aromatase Inhibitor-Resistant Breast Cancer Cells. Breast Cancer Res. Treat..

[11] Matthews H.K., Bertoli C., De Bruin R.A.M. (2022). Cell Cycle Control in Cancer. Nat. Rev. Mol. Cell Biol..

[12] Ducreux M., Abou-Alfa G.K., Bekaii-Saab T., Berlin J., Cervantes A., de Baere T., Eng C., Galle P., Gill S., Gruenberger T. (2023). The Management of Hepatocellular Carcinoma. Current Expert Opinion and Recommendations Derived from the 24th ESMO/World Congress on Gastrointestinal Cancer, Barcelona, 2022. ESMO Open.

[13] Connell L.C., Harding J.J., Shia J., Abou-Alfa G.K. (2016). Combined Intrahepatic Cholangiocarcinoma and Hepatocellular Carcinoma. Chin. Clin. Oncol..

[14] Pauli F.P., Martins J.R., Paschoalin T., Ionta M., Barbosa M.L.C., Barreiro E.J. (2020). Novel VEGFR-2 Inhibitors with an *N*-acylhydrazone Scaffold. Arch. Der Pharm..

[15] Qiu G.-H., Xie X., Xu F., Shi X., Wang Y., Deng L. (2015). Distinctive Pharmacological Differences between Liver Cancer Cell Lines HepG2 and Hep3B. Cytotechnology.

[16] Skehan P., Storeng R., Scudiero D., Monks A., McMahon J., Vistica D., Warren J.T., Bokesch H., Kenney S., Boyd M.R. (1990). New Colorimetric Cytotoxicity Assay for Anticancer-Drug Screening. JNCI J. Natl. Cancer Inst..

[17] Franken N.A.P., Rodermond H.M., Stap J., Haveman J., van Bree C. (2006). Clonogenic Assay of Cells in Vitro. Nat. Protoc..

[18] Melo M.L., Fonseca R., Pauli F., Zavan B., Hanemann J.A.C., Miyazawa M., Caixeta E.S., Nacif J.L.M., Aissa A.F., Barreiro E.J. (2023). *N*-Acylhydrazone Derivative Modulates Cell Cycle Regulators Promoting Mitosis Arrest and Apoptosis in Estrogen Positive MCF-7 Breast Cancer Cells. Toxicol. Vitr..

[19] Pfaffl M.W. (2001). A New Mathematical Model for Relative Quantification in Real-Time RT-PCR. Nucleic Acids Res..

[20] Kassambara A., Kosinski M., Biecek P., Fabian S. Drawing Survival Curves Using “ggplot2”, [R Package Survminer Version 0.4.9]; 2021. https://cran.r-project.org/web/packages/survminer/index.html.

[21] R Core Team (2020). R: A Language and Environment for Statistical Computing.

[22] Ramos M., Geistlinger L., Oh S., Schiffer L., Azhar R., Kodali H., de Bruijn I., Gao J., Carey V.J., Morgan M. (2020). Multiomic Integration of Public Oncology Databases in Bioconductor. JCO Clin. Cancer Inform..

[23] Kalathil D., John S., Nair A.S. (2021). FOXM1 and Cancer: Faulty Cellular Signaling Derails Homeostasis. Front. Oncol..

[24] Nair A.S., Singh A.K., Kumar A., Kumar S., Sukumaran S., Koyiparambath V.P., Pappachen L.K., Rangarajan T.M., Kim H., Mathew B. (2022). FDA-Approved Trifluoromethyl Group-Containing Drugs: A Review of 20 Years. Processes.

[25] Arzumanian V.A., Kiseleva O.I., Poverennaya E.V. (2021). The Curious Case of the HepG2 Cell Line: 40 Years of Expertise. Int. J. Mol. Sci..

[26] Levrero M., Zucman-Rossi J. (2016). Mechanisms of HBV-Induced Hepatocellular Carcinoma. J. Hepatol..

[27] Chen L., Luo L., Chen W., Xu H.-X., Chen F., Chen L.-Z., Zeng W.-T., Chen J.-S., Huang X.-H. (2016). MicroRNA-24 Increases Hepatocellular Carcinoma Cell Metastasis and Invasion by Targeting P53: miR-24 Targeted P53. Biomed. Pharmacother..

[28] Pollutri D., Gramantieri L., Bolondi L., Fornari F. (2016). TP53/MicroRNA Interplay in Hepatocellular Carcinoma. Int. J. Mol. Sci..

[29] Müller-Coan B.G., Caetano B.F.R., Pagano J.S., Elgui De Oliveira D. (2018). Cancer Progression Goes Viral: The Role of Oncoviruses in Aggressiveness of Malignancies. Trends Cancer.

[30] Liu Z., Sun Q., Wang X. (2017). PLK1, A Potential Target for Cancer Therapy. Transl. Oncol..

[31] Willems E., Dedobbeleer M., Digregorio M., Lombard A., Lumapat P.N., Rogister B. (2018). The Functional Diversity of Aurora Kinases: A Comprehensive Review. Cell Div..

[32] Zhao X., Yan H., Yan X., Chen Z., Zhuo R. (2022). A Novel Prognostic Four-Gene Signature of Breast Cancer Identified by Integrated Bioinformatics Analysis. Dis. Markers.

[33] Islam B., Yu H.-Y., Duan T.-Q., Pan J., Li M., Zhang R.-Q., Masroor M., Huang J.-F. (2023). Cell Cycle Kinases (AUKA, CDK1, PLK1) Are Prognostic Biomarkers and Correlated with Tumor-Infiltrating Leukocytes in HBV Related HCC. J. Biomol. Struct. Dyn..

[34] Su W.-L., Chuang S.-C., Wang Y.-C., Chen L.-A., Huang J.-W., Chang W.-T., Wang S.-N., Lee K.-T., Lin C.-S., Kuo K.-K. (2020). Expression of FOXM1 and Aurora-A Predicts Prognosis and Sorafenib Efficacy in Patients with Hepatocellular Carcinoma. Cancer Biomark..

[35] Ahn J.Y., Chung E.Y., Kwun H.J., Jang K.L. (2001). Transcriptional Repression of P21waf1 Promoter by Hepatitis B Virus X Protein via a P53-Independent Pathway. Gene.

[36] Luo X., Zhou N., Wang L., Zeng Q., Tang H. (2019). Long Noncoding RNA GATA3-AS1 Promotes Cell Proliferation and Metastasis in Hepatocellular Carcinoma by Suppression of PTEN, CDKN1A, and TP53. Can. J. Gastroenterol. Hepatol..

[37] Xie M., Sun M., Zhu Y., Xia R., Liu Y., Ding J., Ma H., He X., Zhang Z., Liu Z. (2015). Long Noncoding RNA HOXA-AS2 Promotes Gastric Cancer Proliferation by Epigenetically Silencing P21/PLK3/DDIT3 Expression. Oncotarget.

[38] Khan M.A., Khan P., Ahmad A., Fatima M., Nasser M.W. (2023). FOXM1: A Small Fox That Makes More Tracks for Cancer Progression and Metastasis. Semin. Cancer Biol..

[39] Hu G., Yan Z., Zhang C., Cheng M., Yan Y., Wang Y., Deng L., Lu Q., Luo S. (2019). FOXM1 Promotes Hepatocellular Carcinoma Progression by Regulating KIF4A Expression. J. Exp. Clin. Cancer Res..

[40] Li R., Okada H., Yamashita T., Nio K., Chen H., Li Y., Shimakami T., Takatori H., Arai K., Sakai Y. (2022). FOXM1 Is a Novel Molecular Target of AFP-Positive Hepatocellular Carcinoma Abrogated by Proteasome Inhibition. Int. J. Mol. Sci..

[41] Wang I.-C., Chen Y.-J., Hughes D., Petrovic V., Major M.L., Park H.J., Tan Y., Ackerson T., Costa R.H. (2005). Forkhead Box M1 Regulates the Transcriptional Network of Genes Essential for Mitotic Progression and Genes Encoding the SCF (Skp2-Cks1) Ubiquitin Ligase. Mol. Cell Biol..

[42] Chai N., Xie H., Yin J., Sa K., Guo Y., Wang M., Liu J., Zhang X., Zhang X., Yin H. (2018). FOXM1 Promotes Proliferation in Human Hepatocellular Carcinoma Cells by Transcriptional Activation of CCNB1. Biochem. Biophys. Res. Commun..

