# Supplementary material for: Antiproliferative Activity of N-Acylhydrazone Derivative on Hepatocellular Carcinoma Cells Involves Transcriptional Regulation of Genes Required for G2/M Transition
Source: Biomedicines. 2024 Apr 18;12(4):892. doi: 10.3390/biomedicines12040892 (PMC11048582; doi:10.3390/biomedicines12040892)
Supplement: Supplementary file 1 [file biomedicines-12-00892-s001.zip › Table S1.pdf]

Table S1. Sequences of the primers used for amplification in real-time PCR.

Gene	Sequence	Reference
PLK1	F 5′-CCTGCACCGAAACCGAGTTAT-3’	NM_005030.5
	R 5′-CCGTCATATTCGACTTTGGTTGC-3’	
CDK1	F: 5′-ATGAGGTAGTAACACTCTGG-3’	NM_001786.4
	R: 5′-CCTATACTCCAAATGTCAACTG-3’	
CDKN1A	F 5’- CCATAGCCTCTACTGCCACCATC-3’	NM_001291549.1
	R 5’- GTCCAGCGACCTTCCTCATCCA-3’	
CCNB1	F 5’- GTACCCTCCAGAAATTGGTGA-3’	NM_031966.2
	R 5’- GACTACATTCTTAGCCAGGTG-3’	
AURKA	F 5’- TCTTCACAGGAGGCAAATCCA-3’	NM_198434.3
	R 5’- AATAAGTTACACACTCACTCAGGTACTA-3’	
AURKB	F 5’- AAAGAGCCTGTCACCCCATC-3’	NM_001313950.2
	R 5’- CGCCCAATCTCAAAGTCATC-3’	
CCND1	F 5’- CCATAGCCTCTACTGCCACCATC-3’	NM_001291549.1
	R 5’- GTCCAGCGACCTTCCTCATCCA-3’	
FOXM1	F: 5′-TGCCCAGCAGTCTCTTACCT-3′	NM_001243089.1
	R: 5′-CTACCCACCTTCTGGCAGTC-3′	
ACTB	F 5’- AGAGCTACGAGCTGCCTGAC-3’	NM_001101.3
	R 5’- AGCACTGTGTTGGCGTACAG-3’	

F = forward primer; R = reverse primer
